# Hemodynamic monitoring in Swiss ICUs: results from a Web-based survey

**DOI:** 10.1186/cc9453

**Published:** 2011-03-11

**Authors:** N Siegenthaler, R Giraud, T Saxer, JA Romand, K Bendjelid

**Affiliations:** 1 Hôpital Cantonal Universitaire, Genève, Switzerland

## Introduction

Adequate and prompt implementation of hemodynamic monitoring is an essential component in the management of critically ill patients. The goal of the present survey is to assess hemodynamic monitoring strategies in Swiss ICUs.

## Methods

A self-reported Web-based questionnaire (36 multiple-choice questions) was sent by email to available physicians in charge of adult critically ill patients in Swiss ICUs. The survey examined two subjects: the monitoring tool used and how the clinicians address fluid responsiveness. Results where expressed as frequency (% of all replies) and/or presented as a mean rate.

## Results

We obtained 130 replies from 71% of selected Swiss ICUs (general, surgical, medical, etc.). Devices available were: echocardiography (Echo): 94.5%, PiCCO: 87.3%, Swan-Ganz: 80%, FloTrac™: 21.8%, oesophageal Doppler: 16.4%, LiDCO: 10.9%. The most often device used was: PiCCO: 56.7%, Swan-Ganz: 30.7%, Echo: 8.7%, FloTrac™: 3.1%, LiDCO: 0.8% respectively. Clinicians classified (from 1 to 5) the available devices in various situations as follows: during cardiogenic shock: Swan-Ganz (4.27), Echo (4.26), PiCCO (3.62), FloTrac™ (2.43); during septic shock: PiCCO (4.32), Swan-Ganz (3.76), Echo (3.32), FloTrac™ (2.59); during ARDS: PiCCO (4.09), Swan-Ganz (4.01), Echo (3.39), FloTrac™ (2.4). For most of the clinicians, the targeted arterial blood pressure was: 60 to 65 mmHg for 56.2%, 65 to 70 mmHg: 26.9%, 55 to 60 mmHg: 7.7%, 70 to 75 mmHg: 4.6% respectively. The parameters used to predict fluid responsiveness were: PPV: by 58.5% of clinicians, Echo parameters: 55.8%, passive leg rising (PLR) test: 53.8%, SVV: 50.0%, GEDV: 45.5%, CO: 45.4%, ScVO_2_: 43.1%, systemic arterial pressure: 41.5%, pulmonary artery occlusion pressure (PAOP): 34.6%, EVLW: 33.3%, SVO_2_: 31.9%, central venous pressure: 30.8%, variation of inferior vena cava diameter: 27.5%, ITBV: 21.4%, fluid balance: 14.6%, inferior vena cava diameter: 12.5%. Parameters used to stop the vascular filling were: high EVLW: by 51.8% of clinicians, high PAOP: 50.9%, low PPV: 42.6%, high GEDV: 42.0%, disappearance of lactates: 41.9%, Echo parameters: 39.5%, negative PLR test: 38.0%, high ITBV: 30.4%, increase in oxygen requirement: 25.6%, normal CO: 23.3%, elevated CO: 6.2%, high ScVO_2_: 18.6%, high SVO_2_: 13.3%.

## Conclusions

This study suggests that clinicians use diverse monitoring methods. Moreover, regarding the parameters used for the fluid management strategy, several parameters are used without a clear predominance for one of them. Furthermore, static indices remain used.

